# Surfing in the streets: How problematic smartphone use, fear of missing out, and antisocial personality traits are linked to driving behavior

**DOI:** 10.1371/journal.pone.0284984

**Published:** 2023-04-27

**Authors:** Matthias F. C. Hudecek, Simon Lemster, Peter Fischer, Julia Cecil, Dieter Frey, Susanne Gaube, Eva Lermer

**Affiliations:** 1 Department of Experimental Psychology, University of Regensburg, Regensburg, Germany; 2 LMU Center for Leadership and People Management, LMU Munich, München, Germany; 3 Department of Infection Prevention and Infectious Diseases, University Hospital Regensburg, Regensburg, Germany; 4 Department of Business Psychology, Technical University of Applied Sciences Augsburg, Augsburg, Germany; Private University Schloss Seeburg: Privatuniversitat Schloss Seeburg, AUSTRIA

## Abstract

Smartphone use while driving (SUWD) is a major cause of accidents and fatal crashes. This serious problem is still too little understood to be solved. Therefore, the current research aimed to contribute to a better understanding of SUWD by examining factors that have received little or no attention in this context: problematic smartphone use (PSU), fear of missing out (FOMO), and Dark Triad. In the first step, we conducted a systematic literature review to map the current state of research on these factors. In the second step, we conducted a cross-sectional study and collected data from 989 German car drivers. A clear majority (61%) admitted to using the smartphone while driving at least occasionally. Further, the results showed that FOMO is positively linked to PSU and that both are positively associated with SUWD. Additionally, we found that Dark Triad traits are relevant predictors of SUWD and other problematic driving behaviors––in particular, psychopathy is associated with committed traffic offenses. Thus, results indicate that PSU, FOMO, and Dark Triad are relevant factors to explain SUWD. We hope to contribute to a more comprehensive understanding of this dangerous phenomenon with these findings.

## 1. Introduction

According to a report by the National Highway Traffic Safety Administration, in the US alone 3,142 people were killed, and an estimated 424,000 people were injured in accidents involving distracted drivers in 2019. Four hundred twenty-two persons died in crashes in which at least one driver was distracted by a phone. Examining the age distribution, about 38% of drivers involved in fatal crashes were aged between 15 and 34. At the same time, this group was overrepresented in accidents due to smartphone use at the wheel. About 56% of the involved drivers distracted by their phones were in this age span [1]. Thus, distracted driving due to problematic smartphone use is critical among young people. In a student survey with participants aged 18–29, about 90% reported that they sometimes text while driving, and 50% even text on the highway [2]. A more recent study found that young drivers (17–22 years) touched their smartphones on average 1.71 times per minute while driving [3]. Wilson and Stimpson [4] analyzed data from the Fatality Analysis Reporting System (FARS), a database that includes information on all fatalities occurring on public roads in the United States from 1999 to 2008. FARS provides information on driver-related factors for each accident. Wilson and Stimpson’s study focused on trends in distracted driving fatalities, driver and crash characteristics, as well as cell phone usage and texting volume. They found a link between the number of text messages sent in the US and the percentage of persons killed on the roads due to distracted driving using multivariate regression analyses. Thus, if text message volumes had been zero during the period 2002–2007, predicted fatalities from distracted driving would have declined from 4,611 to 1,925 per year in that time. In fact, in the United States, fatalities increased from 4,611 in 2001 to 5,988 in 2007, which represents a 30% increase. Hill et al. [2] showed that the more often a person drove, the more likely they were to use their phone while driving. Despite the apparent dangers of frequent smartphone use while driving (SUWD), it is not fully understood what influences this behavior [5] and who is particularly prone to it.

We first introduce three psychological constructs that have well-established links with problematic behaviors and are, therefore, good candidates for predicting SUWD: (a) problematic smartphone use; (b) fear of missing out; and (c) Dark Triad personality.

### 1.1. Problematic smartphone use

Problematic smartphone use (PSU) is a phenomenon that has engaged research intensively in recent years [6]. For example, in one survey, nearly 50% of respondents said they could not live without their smartphones [7]. Typically, PSU as a construct refers to the excessive use of smartphones, which negatively impacts various areas of life such as work, school, or social interactions [8]. Research has shown that PSU is associated with adverse outcomes such as anxiety [9]. In addition, PSU can predict engagement in unsafe behaviors such as phone use while driving [10] and dangerous driving [11, 12] Regarding SUWD, there has been consensus on the adverse effects and the danger of this behavior. Not surprisingly, drivers using a phone have a higher risk of crashing [13, 14]. Thus, various studies have examined factors contributing to phone use while driving. It has been shown that older age, having more passengers in the car, and the presence of children are negatively associated with using a phone while driving [15]. In addition, psychosocial factors such as perceived risks and attitudes have been linked to using a phone while driving [16, 17]. Some studies also investigated the relevance of PSU in this context. An Australian study found a link between PSU and drivers’ decision to use a phone while driving [10] Further, Nguyen-Phuoc et al. [18] reported that PSU was the strongest predictor for phone use while driving a car, with attitudes and beliefs being less influential. Another study conducted by Kita and Luria [3] reported that a strong manifestation of PSU was a mediator between personality and phone use while driving.

**Fig 1:**
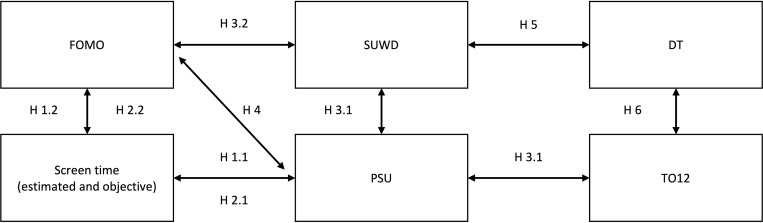
Overview of hypotheses of the study. *Note*. FOMO = Fear of missing out; SUWD = Smartphone use while driving; DT = Dark Triad traits; Screen time (estimated and objective) = Estimated screen time and objective screen time; PSU = Problematic smartphone use; TO12 = Penalized traffic offenses in the last 12 months.

Most studies in the context of PSU or social media use rely on self-reports instead of objectively measured behavior (e.g., [19]), which may be biased due to cognitive misjudgments and socially desirable responding. A recent study investigating PSU, therefore, assessed participants’ screen time using a free Android application to examine smartphone use more objectively [20]. Results indicate that longer daily screen time is the best predictor of PSU. Based on this previous research, we propose the following hypotheses (see also Fig 1):

*H1*.*1* Estimated screen time is positively associated with problematic smartphone use.
*H2*.*1* Objective screen time is positively associated with problematic smartphone use.
*H3*.*1* Problematic smartphone use is positively associated with smartphone use while driving and penalized traffic offenses in the last 12 months.

### 1.2. Fear of missing out

Closely related to PSU is the concept of fear of missing out (FOMO), which McGinnis [21] established as a common social influencing factor on young people’s mental health. One of the first articles that explored FOMO empirically described it as “pervasive apprehension that others might be having rewarding experiences from which one is absent” [22, p. 1]. This apprehension manifests itself in an unwillingness to agree to social commitments to avoid missing out on an even better experience. It may even lead to a state of not committing to any group activities [21]. Although FOMO is a phenomenon occurring across all age groups, younger persons, especially young men, seem to be particularly affected [22]. In general, the concept of FOMO is not restricted to experiences in the virtual world of social networks. Nevertheless, plenty research on FOMO revolves around media, the internet, and smartphone addiction. The feeling of missing out on rewarding experiences is multiplied by the permanent availability of insights on other people’s adventures through platforms like Instagram [23]. In combination with excessive or even addictive internet use, a distorted, overly-positive perception of others can lead to self-doubt [23], decreased subjective well-being [24], and depression [25]. In the latter study, Baker et al. [25] also found correlations between FOMO and perceived somatic symptoms. These were measured using the Physical Symptoms Checklist [26], including conditions like chest pain, headache, or sore throat. Additionally, FOMO was linked to experiencing more negative alcohol-related consequences among college students, ranging from feeling powerless over problems with the family to having a blackout [27]. These alarming findings give rise to further investigation on the negative impact of FOMO on both mental and physical health. Regarding the impact of FOMO in the context of driving, research is relatively scarce. A recent study by Brown et al. [28] has shown that FOMO could predict the probability of sending text messages while driving. In addition, FOMO was associated with longer reaction times when participants were confronted with social reward distractors [29]. Thus, we propose the following hypotheses (see also Fig 1):

*H1*.*2* Estimated screen time is positively associated with fear of missing out.
*H2*.*2* Objective screen time is positively associated with fear of missing out.
*H3*.*2* Fear of missing out is positively associated with smartphone use while driving.

In addition, we hypothesize positive associations between PSU and FOMO:

*H4* Fear of missing out is positively associated with problematic smartphone use.

### 1.3. Dark Triad

The term Dark Triad was first established by Paulhus and Williams in 2002 [30], and it refers to three personality traits that feature non-pathologic malicious social behavior: (a) subclinical psychopathy, (b) subclinical narcissism, and (c) Machiavellianism. Plenty of research has shown associations between all three traits and job-related outcomes, personal life, and relationships [31]. In addition, narcissism and psychopathy have been linked to problematic driving behavior [32]. Panayiotou [33] found correlations between lack of empathy and self-reported driving violations, but the study only included men in military service and no women. Haapasalo [34] conducted a study examining the types of offenses committed by convicted criminals and found that non-psychopaths (89%) were more often sentenced for traffic offenses than (clinical) psychopaths (83%). However, this difference was not statistically significant. Links between narcissism and problematic driving behavior have also been found in the past, but mainly in the context of anger and aggressive driving [35]. It has been shown that both men and women with narcissism are more likely to react aggressively and vengefully towards perceived wrongdoings of others in traffic. To the best of our knowledge, no studies have examined the association between Machiavellianism and problematic driving behavior so far, nor how Machiavellianism interacts with smartphone use at the wheel. In their review, Fehr et al. [36] pointed out that manipulative behavior is a key correlate of Machiavellianism. In addition, persons scoring high on Machiavellianism cheat when the chance of getting caught is low [37]. Consequently, it is possible that people scoring high on Machiavellianism might “cheat” (i.e., bending or breaking rules) in traffic. Therefore, this study aims to close this research gap, and we expect Machiavellianism as well as narcissism and psychopathy to be associated with problematic driving behaviors:

*H5*. All Dark Triad traits are positively associated with smartphone use while driving.
*H6*. All Dark Triad traits are positively associated with penalized traffic offenses in the last 12 months.

Since there is little literature on the relationship between the Dark Triad traits and PSU or driving behaviors, we conducted a systematic literature review to determine the extent of these research gaps.

### 1.4. Literature review

To further explore the association of SUWD, PSU, FOMO, and Dark Triad, we conducted a systematic literature search. We searched databases (Web of Science, PsycINFO, PsycARTICLES) for the keywords smartphone AND driving AND problematic smartphone use OR fear of missing out OR Dark Triad. This search led to 454 results. Abstracts were then screened for relevance. Studies were excluded when the focus was not on the relationship between our variables of interest, e.g., studies that evaluated the driving style based on data from smartphone sensors. After removing studies that met our exclusion criteria and deleting duplicates, 11 studies remained. These articles were then reviewed in more detail. Since the present study focuses on the association between SUWD, FOMO, and Dark Triad studies only looking at the relationships between smartphone use and other variables (e.g., drivers’ acceptance of technology that reduces mobile phone use while driving), were also excluded. None of the remaining studies examined Dark Triad specifically, which means there is no previous research on the associations between Dark Triad and SUWD. Therefore, we decided to include studies on more general personality traits such as Big Five. This resulted in seven relevant studies (see Table 1).

**Table 1:** Overview of studies investigating the relationship between smartphone use while driving, problematic smartphone use, fear of missing out, and personality traits.

			Sample					
Reference	Design	Size	Features	Location	Central measures	Observation concerning PSU	FOMO	P
Kaviani et al. [40]	Cross-sectional (online survey)	2,774	Mostly between 40 and 59 years, 53% female, Victoria residents with a valid driver’s license	Australia	Smartphone use while driving, nomophobia	• Longer average time spent on smartphones was one of the strongest positive predictors of smartphone use while driving • Not being able to access information was the only nomophobia factor that significantly predicted smartphone use while driving	x	
Matias et al. [29]	Driving visual search task	29	*M*_age_ = 20, 75.9% female, college students	France	FOMO, smartphone, distraction, social reward	• Individuals‘ level of FOMO can predict the distraction triggered by high social reward stimuli in a driving context	x	
Yeo and Park [61]	Cross-sectional (questionnaire)	948	*M*_age_ = between 20 and 50, 31% female, 739 online and 209 offline participants	South Korea	Smartphone use, Perceived risk, Smartphone dependency	• Higher smartphone dependency was associated with a higher smartphone use while driving	x	
Kita and Luria [3]	Cross-sectional (online questionnaire & app)	221	*M*_age_ = 19.3, 35.3% female, driver’s license for at least 3 months	Israel	Big Five, Smartphone addiction, smartphone use while driving	• Positive relationship between extraversion and neuroticism and smartphone use while driving • Smartphone addiction mediates the relationship between neuroticism (but not extraversion) and smartphone use while driving		x
Maier et al. [5]	Cross-sectional (questionnaire)	273	*M*_age_ = 28.5, 39.7% female, members of the ADAC	Germany	Smartphone use while driving, Big Five, personality profiles	• Drivers of the three personality profiles (non-neurotic driver, extraverted-open driver, and conscientious driver) are more likely to use their smartphone while driving		x
Nguyen-Phuoc et al. [62]	Cross-sectional (questionnaire)	857	Mostly between 18 and 25 years, 529 motorcyclists, 328 car drivers	Vietnam	Perceived risk, problematic mobile phone use, frequency of mobile phone use while driving, attitudes and beliefs	• Differences between motorcyclists and car drivers: • For both groups: PSU and attitudes and beliefs had significant positive impacts on the frequency of smartphone use • Motorcyclists: most substantial effect of attitudes and beliefs on smartphone use • Car drivers: strongest effect of PSU on smartphone use		x
Zhang et al. [38]	Cross-sectional (questionnaire)	317	*M*_age_ = 28.69, 5.5% female, delivery men	China	Driving self-efficacy, mobile phone use while driving, personality traits	• Higher levels of psychoticism were significantly associated with a higher frequency of mobile phone use while driving • Driving self-efficacy had a mediating effect on the relation between psychoticism and mobile phone use while driving		x

*Note*. PSU = problematic smartphone use, FOMO = fear of missing out, P = personality.

As none of the studies examined the Dark Triad traits, studies examining more general personality traits (e.g., Big Five) were also included.

The literature review shows that the combination of smartphone use and driving with FOMO, PSU or Dark Triad has not been studied before. However, three studies have looked more closely at FOMO and SUWD and four other studies examined the relevance of personality in the context of problematic driving behavior and smartphone use. Regarding FOMO and driving, research has shown that FOMO can predict how distracted a driver is while driving [29]. Furthermore, SUWD increases when a person experiences a high fear of being without their smartphone (Kaviani et al., 2020).

Previous studies analyzed associations between individual personality traits such as extraversion and driving behavior on the one hand (e.g., [3]) and the association between personality *profiles* and driving behavior on the other hand [5]. Here, the literature review yielded mixed results. In particular, one article showed that high levels of extraversion are positively related to SUWD. Furthermore, positive associations were found between SUWD and neuroticism [3]. However, in a more recent study, there were no direct effects of extraversion on phone use while driving [38]. The same study also reported that the factor driving self-efficacy plays an essential role as it mediated the positive relationship between psychoticism and problematic SUWD. Regarding different personality profiles, Maier et al. [5] identified three profiles that predict frequent SUWD. Their results indicate that a profile perspective might be more beneficial than focusing on single traits. For example, neuroticism is low in the profiles of the *non-neurotic driver* but high in the *conscientious driver*. Thus, a specific trait (e.g., neuroticism) can be either low or high but can still equally contribute to problematic SUWD in a particular constellation with other traits. This highlights the importance of more integrative models, which require different perspectives on the dangerous phenomenon of SUWD.

In summary, research on PSU, FOMO, and personality in the context of SUWD is scarce. This is surprising considering the high number of people killed in accidents each year caused by distraction due to SUWD. We found no study that analyzed the relevance of PSU or FOMO *and* personality for driving behavior. In addition, no previous research has been conducted examining the association between Dark Triad personality traits and SUWD. The findings from Maier et al. [5] stress the importance of developing more integrative models for understanding complex human behavior such as SUWD. Focusing on the Dark Triad and FOMO––so far, underexplored factors that might explain SUWD––can be seen as one step towards such a comprehensive understanding.

## 2. Method

### 2.1. Sample

Participants were recruited mainly through mailing lists and social networks, such as Facebook. Apart from being over 18 years of age, requirements for participating in the study were smartphone ownership and regular driving. Informed consent was obtained by all participants prior to participation. The study did not need approval by the ethics boards according to the standards of the University of Regensburg as the survey was no experimental study. The participants did not belong to a vulnerable group of people nor was the study expected to cause any form of psychological or physical distress, or anxiety. A confirmation of the ethics committee of our university that no vote of the ethics committee was required to conduct this study is available.

The study sample consisted of 989 participants, with 724 (73%) women, 263 (26%) men, and two participants with other sexual identities. The mean age was 26.17 years (*SD* = 8.09 years). While 947 (95%) subjects had German citizenship, 42 (5%) did not. Of all participants, 165 (17%) had a university degree, 463 (57%) had attained the university entrance qualification, 230 (23%) had completed vocational training, and 31 (3%) held an educational level lower than that. Regarding their professional status, 422 (43%) participants were employees, freelancers, or managing directors, 503 (51%) were students, and one participant attended high school. The other 63 (6%) subjects had other occupations or none at all. Power analysis indicates this sample size is sufficient to detect a small effect size, f2(.02), at 95% power (given α = .05 and four predictors).

### 2.2. Measures

#### 2.2.1. Car usage and driving behavior

To assess the participants’ car usage, we included several questions before the main part of the questionnaire started. First, participants were asked how often they drive a car, rated on a 7-point scale (1 = *never*, 7 = *daily*). This also served as a filter question to exclude subjects who did not drive regularly. Then, participants were asked if they own a driver’s license, and if confirmed, we asked whether they were still on probation. Additionally, car ownership was surveyed. Finally, the participants were asked whether they had committed a traffic offense within the last 12 months, for which they received a penalty. Previous studies indicate that self-reports are suitable and valid for estimating the incidence of traffic offenses of young drivers [39].

#### 2.2.2. Smartphone use while driving (SUWD)

To the best of the authors’ knowledge, no validated instrument existed at the time of data collection to capture SUWD. Therefore, measures from other studies [e.g., 40] were adapted to the context of the current study: Participants were asked if they at least occasionally use their smartphones while driving. If this was affirmed, six explorative items on SUWD followed for a more detailed investigation into the behavior (see Table 2 for an overview of the items). Three out of the six items focused on compensatory strategies [e.g., 41, 42] used during distracted driving (items 3 to 5). All items were rated on a 7-point scale (1 = *do not agree at all*, 7 = *totally agree*). As all of these items captured unidimensional and narrow behaviors, the use of single-item measures was considered appropriate [43, 44]. Most research on single-item measures shows that they are usually as valid and reliable as multiple-item measurement instruments [45, 46].

**Table 2:** Overview of the six items used to measure smartphone use while driving (SUWD).

Item no.	German version (Original)	English version
1	Wenn beim Autofahren Langeweile aufkommt, beschäftige ich mich mit meinem Smartphone.	When I get bored while driving, I spend time on my smartphone.
2	Wissentlich emotionale Telefonate führe ich auch während der Autofahrt.	I make emotional phone calls while driving.
3	Wenn ich mein Smartphone während der Fahrt benutze, versuche ich zeitgleich öfter die Spiegel zu überprüfen.	When I use my smartphone while driving, I try to check my mirrors more often at the same time.
4	Wenn ich mein Smartphone während der Fahrt benutze, halte ich zeitgleich mehr Abstand zum vorderen Fahrzeug.	When I use my smartphone while driving, I simultaneously keep more distance from the vehicle in front.
5	Wenn ich mein Smartphone während der Fahrt benutze, versuche ich es möglichst unauffällig zu verwenden, sodass es andere Verkehrsteilnehmer nicht sehen können.	When I use my phone while driving, I try to use it as discreetly as possible so that other road users cannot see it
6	Wenn ich mein Smartphone während der Fahrt benutze und bemerke, dass andere Verkehrsteilnehmer mich beobachten, fühle ich mich ertappt.	When I use my phone while driving and notice other road users are watching me, I feel caught

*Note*. All items were rated on a 7-point scale (1 = *do not agree at all*, 7 = *totally agree*).

#### 2.2.3. Smartphone use and screen time

Participants were asked about their smartphone brand to assess if they use an Apple iOS device, which was relevant for screen time assessment. We used a feature of iPhones (from version iOS 12) to examine the screen time, equipped with an integrated application automatically collecting screen time data (total screen time and screen time per category, e.g., social media). Therefore, iPhone users were asked to open this application and report the 7-day screen time values, both the total value and the value for the social media category. Additionally, all participants were asked to estimate their screen time. Participants who used a smartphone from a different brand than Apple were directly asked for their screen time estimate. If they also used a screen time tracking app, they had the opportunity to report their objective values. These different measures might have led to varying values. However, this method was still the best option because an on-site evaluation was not possible within this online survey [e.g., 47].

#### 2.2.4. Problematic smartphone use (PSU)

We used the Short Version of the Problematic Mobile Phone Use Questionnaire (PMPUQ-SV) by Lopez-Fernandez et al. [48] to measure problematic smartphone use (PSU). The PMPUQ-SV consists of 15 items rated on a 4-point scale (1 = *strongly agree*, 4 = *strongly disagree*). This version contains all five items of the *dangerous use*-subscale, all five items of the *prohibited use*-subscale, and five of the seven items of the *dependence*-subscale, all from the original PMPUQ-version [49]. All items were averaged to an index of PSU (Cronbach’s α = .82, 95% CI [.80, .84]).

#### 2.2.5. Fear of missing out (FOMO)

To assess FOMO, we used a German adaptation [50] of the Fear of Missing Out Scale (FoMOS) by Przybylski et al. [22]. The scale consists of 10 items about the subjects’ everyday experience and are rated on a 5-point scale (1 = *not at all true of me*, 5 = *extremely true of me*). Cronbach’s alpha of the scale was α = .76, 95% CI [.74, .78].

#### 2.2.6. Dark Triad (DT)

Dark Triad personality traits were measured using the German version [51] of the Short Dark Triad (SD3) by Jones and Paulhus [52]. The SD3 contains 27 items covering the subscales Machiavellianism, narcissism, and psychopathy. All items are rated on a 5-point scale (1 = *strongly disagree*, 5 = *strongly agree*). Cronbach’s Alpha ranged from α = .69 to .71 (Machiavellianism: 95% CI [.75, .79]; narcissism: 95% CI [.68, .73]; psychopathy: 95% CI [.66, .72]).

All measures described above are openly available in the Open Science Framework repository of this article at https://osf.io/eup63.

### 2.3. Analyses

Statistical analysis was performed with R Studio version 1.4.1106. Statistical analysis involved descriptive and correlative analysis as well as linear and logistic regression. Assumptions and prerequisites were checked. Unless stated otherwise, all inference statistical analyses were performed at a significance level of α = .05. We analyzed objective and estimated screen time separately to achieve the most reliable results. In addition, we rounded all reported screen times to the last full hour to ensure a uniform data structure. In order to reduce the probability of type I error and to limit it to a maximum of α = .05 for the overall hypothesis, a Bonferroni-Holm-correction was subsequently performed [53]. The resulting corrected p-values of all hypotheses remained below the value of *p* = .05 (data and R script can be found in the supplementary material, https://osf.io/eup63).

## 3. Results

The distribution of Apple iOS and other operating systems across the sample was almost split in half (468x iPhone, 289x Samsung (Android), 174x other brands (Android), 7x Windows phone, 51x other). A majority of 600 participants (61%) admitted to using the smartphone while driving at least occasionally. 167 participants (17%) reported committing a penalized traffic offense within the past 12 months.

An overview of the correlations between FOMO, PSU, and screen times can be found in Table 3 (hypotheses 1, 2, and 4). As expected, there was a positive association between FOMO and PSU (confirming H4). We also found a positive correlation between *estimated* screen time and FOMO and PSU (confirming H1). However, there were no associations between *objective* screen time and FOMO and PSU. Exploratory analyses revealed different patterns between iOS and non-iOS users regarding the correlations with estimated and objective screen time. While there were no associations between PSU/FOMO and objective screen time for non-iOS users, we found a small positive correlation between PSU and objective screen time for iOS users (*r* = .18, *p* < .001, 95% CI [.08, .28]). No associations can be reported between FOMO and objective screen time for both operating systems (iOS: *r* = .07, *p* = .160, 95% CI [-.03, .17]; non-iOS: *r* = .05, *p* = .602, 95% CI [-.14, .24]). Therefore, our results only partially confirmed hypothesis 2, stating that objective screen time is positively correlated with FOMO/PSU. Regarding estimated screen time, positive associations between PSU and screen time were found for both operating systems (non-iOS: *r* = .20, *p* < .001, 95% CI [.12, .29]; iOS = *r* = .22, *p* = .010, 95% CI [.05, .37]), whereas there only was a positive correlation for non-iOS users (*r* = .23, *p* < .001, 95% CI [.15, .31]), but not for iOS users (*r* = .09, *p* = .281, 95% CI [-.08, .26]) regarding FOMO.

**Table 3:** Pearson correlations between fear of missing out (FOMO), problematic smartphone use (PSU), and estimated/objective screen time.

	M	SD	1	2
1. FOMO	2.49	0.58		
2. PSU	2.13	0.41	.24**	
			[.18, .29]	
3. Estimated screen time	9.86	10.74	.23**	.20**
			[.15, .31]	[.12, .29]
4. Objective screen time	7.20	9.80	.05	.02
			[-.14, .24]	[-.17, .21]

*Note*. *M* and *SD* are used to represent mean and standard deviation, respectively. Values in square brackets indicate the 95% confidence interval for each correlation. The confidence interval is a plausible range of population correlations that could have caused the sample correlation [63].

* indicates *p* < .05.

** indicates *p* < .01.

FOMO = fear of missing out; PSU = problematic smartphone use.

Regarding the association between PSU as well as FOMO and SUWD (H3), we found moderate correlations (see Table 4). The most substantial relationship was found between PSU and SUWD item 1 (“When I get bored while driving, I spend time on my smartphone”, *r* = .48, *p* < .001, 95% CI [.41, .54]) followed by SUWD item 2 (“I make emotional phone calls while driving.”, *r* = .37, *p* < .001, 95% CI [.30, .44]). We found no correlation between SUWD item 3 (“When I use my smartphone while driving, I try to check my mirrors more often at the same time.”) and SUWD item 4 (“When I use my smartphone while driving, I simultaneously keep more distance from the vehicle in front.”) and PSU as well as FOMO. Additionally, smaller correlations between SUWD item 5 (“When I use my smartphone while driving, I try to use it as discreetly as possible so that other road users can’t see it”) and FOMO (*r* = .15, *p* < .001, 95% CI [.07, .22]) and between SUWD item 6 (“When I use my smartphone while driving and notice other road users watching me, I feel caught.”) and FOMO (*r* = .16, *p* < .001, 95% CI [.08, .24]) were found. Furthermore, a weak association was found between SUWD item 6 and PSU (*r* = .10, *p* = .017, 95% CI [.02, .18]).

**Table 4:** Pearson correlations between fear of missing out (FOMO), problematic smartphone use (PSU), and smartphone use while driving (SUWD).

Variable	*M*	*SD*	1	2	3	4	5	6	7
1. FOMO	2.49	0.58							
2. PSU	2.13	0.41	.24**						
			[.18, .30]						
3. SUWD item 1	2.56	1.76	.12**	.48**					
			[.04, .20]	[.41, .54]					
4. SUWD item 2	2.60	1.72	.08*	.37**	.28**				
			[.00, .16]	[.30, .44]	[.21, .35]				
5. SUWD item 3	4.12	1.86	.07	.00	.10*	.06			
			[-.01, .15]	[-.08, .08]	[.02, .18]	[-.02, .14]			
6. SUWD item 4	5.26	1.46	.02	-.07	-.05	-.04	.33**		
			[-.06, .10]	[-.15, .01]	[-.13, .03]	[-.12, .04]	[.26, .40]		
7. SUWD item 5	5.21	1.63	.15**	.07	-.01	.02	.20**	.17**	
			[.07, .22]	[-.01, .15]	[-.09, .07]	[-.06, .10]	[.12, .28]	[.09, .24]	
8. SUWD item 6	4.46	1.94	.16**	.10*	.02	.02	.13**	.10*	.42**
			[.09, .24]	[.02, .18]	[-.06, .10]	[-.06, .10]	[.05, .20]	[.02, .18]	[.35, .49]

*Note*. *M* and *SD* are used to represent mean and standard deviation, respectively. Values in square brackets indicate the 95% confidence interval for each correlation. The confidence interval is a plausible range of population correlations that could have caused the sample correlation [63].

* indicates *p* < .05.

** indicates *p* < .01.

FOMO = fear of missing out; PSU = problematic smartphone use; SUWD = smartphone use while driving.

SUWD item 1 “When I get bored while driving, I spend time on my smartphone.”; SUWD item 2 “I make emotional phone calls while driving.”; SUWD item 3 “When I use my smartphone while driving, I try to check my mirrors more often at the same time.”; SUWD item 4 “When I use my smartphone while driving, I simultaneously keep more distance from the vehicle in front.” SUWD items 5 “When I use my smartphone while driving, I try to use it as discreetly as possible so that other road users can’t see it.”; SUWD item 6 “When I use my smartphone while driving and notice other road users watching me, I feel caught.”.

All correlations between Dark Triad personality traits and SUWD (H5) can be found in Table 5. While there were small to medium associations between all three Dark Triad traits and SUWD (items 1 and 2), only Machiavellianism was positively associated with one of the compensatory behaviors, SUWD item 5 (“When I use my smartphone while driving, I try to use it as discreetly as possible so that other road users can’t see it”). In addition, there were weak negative associations between the feeling of being caught (“When I use my smartphone while driving and notice other drivers are watching me, I feel caught”) and narcissism (*r* = -.09, *p* = .03, 95% CI [-.17, -.01]), as well as psychopathy (*r* = -.14, *p* < .001, 95% CI [-.22, -.06]). Thus, results support hypothesis 5.

**Table 5:** Pearson correlations between Dark Triad personality traits and smartphone use while driving (SUWD).

Variable	*M*	*SD*	1	2	3	4	5	6	7	8
1. Narcissism	2.70	0.57								
2. Psychopathy	2.12	0.56	.37**							
			[.31, .42]							
3. Machiavellianism	2.94	0.63	.30**	.51**						
			[.24, .36]	[.46, .56]						
4. SUWD item 1	2.56	1.76	.14**	.22**	.19**					
			[.06, .21]	[.14, .30]	[.11, .27]					
5. SUWD item 2	2.60	1.72	.19**	.23**	.17**	.28**				
			[.11, .27]	[.15, .31]	[.09, .25]	[.21, .35]				
6. SUWD item 3	4.12	1.86	.03	.00	.07	.10*	.06			
			[-.05, .11]	[-.08, .08]	[-.01, .15]	[.02, .18]	[-.02, .14]			
7. SUWD item 4	5.25	1.46	.00	-.07	.07	-.05	-.04	.33**		
			[-.08, .08]	[-.15, .01]	[-.01, .15]	[-.13, .03]	[-.12, .04]	[.26, .40]		
8. SUWD item 5	5.21	1.63	-.03	.02	.16**	-.01	.02	.20**	.17**	
			[-.11, .05]	[-.06, .10]	[.08, .24]	[-.09, .07]	[-.06, .10]	[.12, .28]	[.09, .24]	
9. SUWD item 6	4.47	1.94	-.09*	-.14**	-.01	.02	.02	.13**	.10*	.42**
			[-.17, -.01]	[-.22, -.06]	[-.09, .07]	[-.06, .10]	[-.06, .10]	[.05, .20]	[.02, .18]	[.35, .49]

*Note*. *M* and *SD* are used to represent mean and standard deviation, respectively. Values in square brackets indicate the 95% confidence interval for each correlation. The confidence interval is a plausible range of population correlations that could have caused the sample correlation [63].

* indicates *p* < .05.

** indicates *p* < .01.

SUWD = smartphone use while driving.

SUWD item 1 “When I get bored while driving, I spend time on my smartphone.”; SUWD item 2 “I make emotional phone calls while driving.”; SUWD item 3 “When I use my smartphone while driving, I try to check my mirrors more often at the same time.”; SUWD item 4 “When I use my smartphone while driving, I simultaneously keep more distance from the vehicle in front.” SUWD items 5 “When I use my smartphone while driving, I try to use it as discreetly as possible so that other road users can’t see it.”; SUWD item 6 “When I use my smartphone while driving and notice other road users watching me, I feel caught.”.

**Fig 2:**
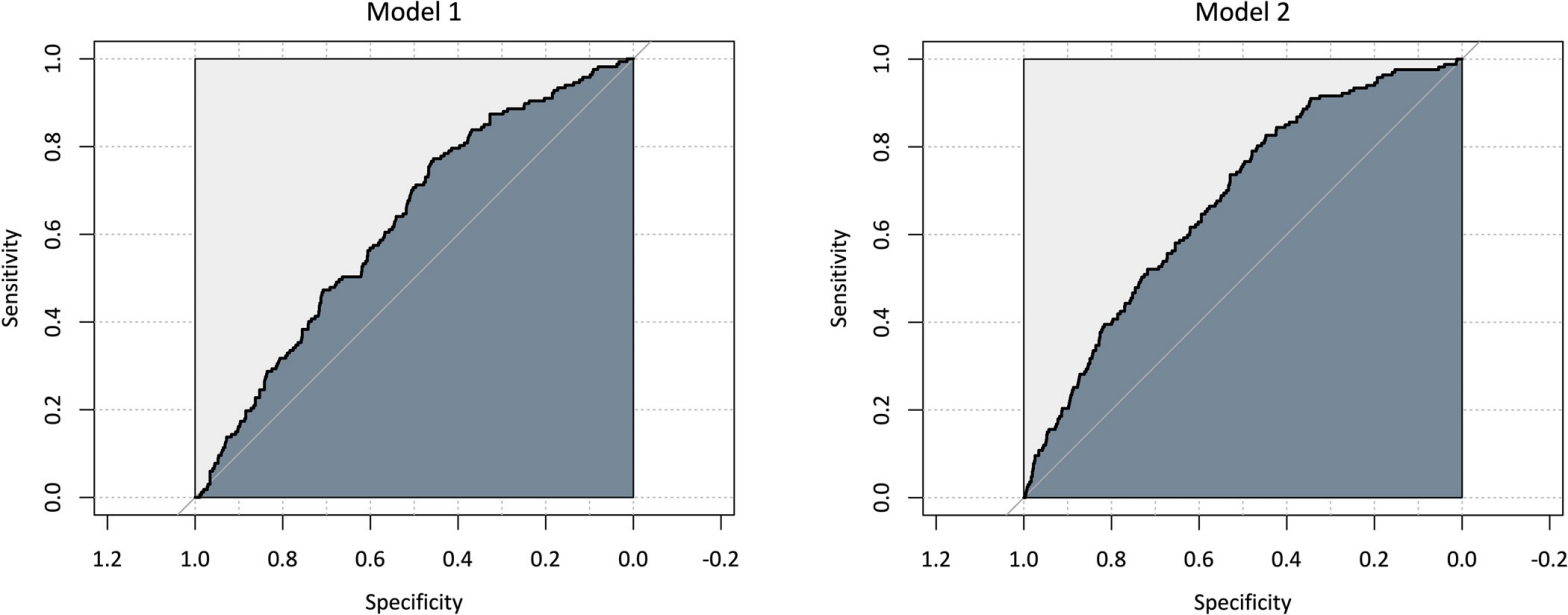
ROC curves for the logistic regression models predicting traffic offense committed (yes/no). *Note*. Model 1: AUC = .625; Model 2: AUC = .675; AUC = area under curve.

To explore Hypothesis 6 (the higher the Dark Triad traits of a person, the more likely they have committed a penalized traffic offense within the last 12 months), we first conducted a logistic regression with the Dark Triad traits as predictors and traffic offense committed (yes / no) as the outcome variable. The model revealed that only psychopathy is a relevant predictor for the outcome variable. The overall model fit (see Table 6) was relatively small, but it had a significantly higher fit than the null model, χ^2^(3) = 22.26, *p* < .001. Based on the point estimates of the model, prognoses can be made: For a person with the minimum psychopathy value of 1 in our sample, the model predicts a probability of 9.89% that this person has committed a traffic offense in the last 12 months. For our sample’s maximum psychopathic value of 4.33, a probability of 56.61% is predicted. Following the steps of Sauer [54], we calculated sensitivity and specificity to further assess the model fit (AUC = .625, see Fig 2). Thus, our results partly support hypothesis 6. Next, we added PSU as another predictor and found that the model fit increased (see Table 6). Again, from the Dark Triad traits, only psychopathy was a relevant predictor for committing a penalized traffic offense within the last 12 months. Moreover, PSU was also revealed to be a substantial predictor. Comparing the log odds between psychopathy and PSU, the latter has a stronger statistical effect. In addition, the AUC of this model improved (AUC = .675). Based on the point estimates of the second model, prognoses can be made: For a person with the minimum psychopathy value of 1 and the minimum PSU value of 1 in our sample, the model predicts a probability of 4.66% that this person has committed a traffic offense in the last 12 months. In contrast, for the maximum psychopathic value of 4.33 and the maximum PSU value of 3.53 in our sample, a probability of 84.77% is predicted.

**Table 6:** Logistic regressions for Dark Triad and problematic smartphone use predicting the odds of a penalized traffic offense in the last 12 months.

Variable	Model 1							Model 2						
						95% CI for OR							95% CI for OR	
	*b*	*SE*	Wald	*p*	OR	Lower	Upper	*B*	*SE*	Wald	*p*	OR	Lower	Upper
Constant	-2.95	0.52	-5.68	< .001				-4.71	0.64	-7.37	< .001			
Narc	0.12	0.17	0.75	.46	1.13	0.82	1.56	0.06	0.17	0.34	.736	1.06	0.76	1.48
Psych	0.74	0.18	4.08	< .001	2.10	1.47	3.01	0.57	0.19	3.01	.003	1.76	1.22	2.55
Mach	-0.20	0.16	-1.27	.206	0.81	0.60	1.12	-0.25	0.16	-1.56	.118	0.78	0.56	1.07
PSU								1.13	0.22	5.10	< .001	0.32	2.00	4.76
*R* ^*2*^_*L*_	.025							.055						
*ΔR* ^*2*^_*L*_								.030						
*R* ^*2*^_*CS*_	.022							.048						
*ΔR* ^*2*^_*CS*_								.026						
*R* ^*2*^_*N*_	.037							.081						
*ΔR* ^*2*^_*N*_								.044						

*Note*. Narc = Narcissism; Psych = Psychopathy; Mach = Machiavellianism; PSU = problematic smartphone

use; OR = odds ratio; *R*^*2*^_*L*_ = Hosmer and Lemeshow’s *R*^*2*^; *R*^*2*^_*CS*_ = Cox and Snell’s *R*^*2*^; *R*^*2*^_*N*_ = Nagelkerle’s *R*^*2*^.

## 4. Discussion

The present study investigated the relationships between fear of missing out (FOMO), problematic smartphone use (PSU), Dark Triad personality traits, and smartphone use while driving (SUWD). Three major findings emerge from this research:

First, we could replicate the positive association between FOMO and PSU found in previous research (confirming hypothesis 4). Thus, this study adds to a broad spectrum of findings identifying connections between FOMO and problematic or even addictive smartphone, social media and internet use [19, 24]. Moreover, our results extend previous findings as we have linked FOMO and PSU with SUWD. We found that PSU, in particular, was positively associated with SUWD, whereas the relationships with FOMO were weaker. No associations were found for both PSU and FOMO with compensatory behaviors of using a phone while driving (such as mirror checking). PSU rather refers to a set of user behaviors that people will even display when driving a car. Thus, a person with higher levels of PSU will more likely use their phone when driving (e.g., to reduce boredom). From a practical perspective, special training and educational programs should be developed to raise awareness about the dangers of PSU and SUWD and to provide them with a set of user behavior change techniques to reduce these problematic behaviors. A review by Sezgin and Lin [55] suggests that using social influence techniques in interventions and parental involvement for promoting safe driving behavior are highly effective. For example, Simons-Morton et al. [56] examined the effectiveness of an in-vehicle safety monitoring system. They could show that a system that gives immediate feedback about risky driving combined with parental involvement had a large effect and yielded a decrease in critical events.

Second, we found mixed results regarding the associations between estimated vs. objectively measured screen time and FOMO as well as PSU. The hypothesized relationships were tested in two ways: on the one hand with objective screen time values derived from smartphone apps, and on the other hand, with self-report estimations. The analysis showed that the self-estimated screen time correlates positively with FOMO and PSU (confirming hypothesis 1). However, there were no associations between the objective screen time and FOMO as well as PSU. Yet, a more differentiated analysis of the sample revealed that this result depended on the type of operating system used by the participants. For iOS users but not users of other operating systems, we found a correlation between objective screen time and PSU (hypothesis 2 partly confirmed). Interestingly, non-iOS users reported higher self-estimated screen time values than iOS users, although their objective screen time was actually lower. These exploratory analyses indicate that there might be behavioral differences between iOS users and users of other operating systems. One potential explanation for these variations might be that the design and functionality of different smartphone brands and operating systems are associated with user behaviors. Our results would indicate that Apple smartphones might increase PSU compared to other phones. In fact, there is some evidence that smartphone addiction might be higher on iOS than Android devices [57]. Another explanation might be that the screen time values provided by smartphones are related to the devices’ brands. There have been reports online about problems with screen time measurement applications [58]. Future research should address this topic in more depth by replicating our findings and evaluating potential explanations for the different screen time values. Regardless of the mixed results, using the smartphones’ integrated data to assess screen time more objectively has proven effective. As screen time tracking is now a feature in almost all smartphones, future research should rely on this type of data more often. Additionally, it would be useful to ask to what extent the participant has already dealt with the topic of PSU.

Third, according to our review, the present study is the first to link Dark Triad personality traits and driving behaviors in the context of PSU. All three traits were positively associated with some forms of SUWD behaviors. Thus, people with Dark Triad personalities tend to use their phones more often while driving. Whereas narcissism and psychopathy were negatively associated with feeling guilty for these kinds of problematic driving behaviors, Machiavellianism was positively related to compensatory behaviors such as more frequent mirror checking. The higher a person scores on Machiavellianism, the more likely they try to use their phone as inconspicuously as possible so that others cannot see it. These findings fit well into previous research on Dark Triad [e.g., 31]. In addition, we found that psychopathy is a useful predictor for previously committed and penalized traffic offenses (confirming hypothesis 6). In our sample, a person with an average level of psychopathy has a 24% probability of having committed a penalized traffic offense in the last 12 months. This compares to a 19% chance for a person with low levels of psychopathy (one standard deviation below mean) and 31% for a person with high levels of psychopathy (one standard deviation above mean). If the psychopathy score in our sample increases by 1 point (on a 5-point scale), the probability of having committed a traffic offense rises to almost 37%. Consequently, psychopathy is a good predictor of traffic offenses.

### 4.1 Limitations

The present study has some limitations. As with all online questionnaires, the collected data are based on self-reports. This means that socially desirable responses cannot be ruled out, even when trying to keep them as minimal as possible by asking about objective criteria, such as the hours spent on social media. In addition, we used a cross-section design which limits causal conclusions based on our results. Still, a causal link between the observed associations would be plausible based on previous findings in the literature. Therefore, the present results can at least be of indicative value to start approaching cause-effect relationships [59]. Nevertheless, future research should use more measurement points and include ratings from other perspectives (e.g., peers).

Although our sample comprised almost 1,000 participants, the gender distribution was not even, as more than 70% were female. This could have affected our results because gender differences in the Dark Triad traits have been observed regularly [e.g., 31, 52] and were also present in our study. Men scored higher on psychopathy, whereas women had higher values on narcissism and Machiavellianism. Men committed two-thirds of the reported traffic offenses. This result is consistent with statistics from the German Federal Motor Transport Authority. The figures from 2020 show that men cause around three to four times more traffic violations than women [60].

In addition, the questionnaire only asked if a penalized traffic offense was committed in the last 12 months, but how many of them. Therefore, participants who were found to have committed a traffic offense once in 12 months were treated the same way as people who violated the rules more frequently. Thus, future investigations should also examine the exact number of penalized offenses and include them in the statistical analyses. Moreover, our data do not provide information about the severity of the committed traffic offenses. Therefore, it is imaginable that the seriousness of the offenses committed varies depending on the psychopathy value.

## 5. Conclusion

Overall, the present results provide relevant insights into the context of smartphone use while driving. To date, the interventions developed and implemented to reduce SUWD have proven to be relatively ineffective. Given the importance of this topic, it is of great practical relevance to better understand which factors contribute to this dangerous behavior. Here, our study can contribute from a theoretical and practical perspective by pointing out the relevance of PSU and the Dark Triad personality traits to explain SUWD. Overall, PSU is an excellent predictor regardless of the Dark Triad personality traits. Since this factor can be changed more easily than personality, PSU should be targeted in public safety interventions, driving training, and court-mandated medical-psychological assessment of driver fitness. It might be a good strategy to help people reduce their PSU in everyday life, which should indirectly decrease the chances of using their phones on the road and prevent accidents and fatal crashes.
